# Water‐Based Solution Processing and Wafer‐Scale Integration of All‐Graphene Humidity Sensors

**DOI:** 10.1002/advs.201802318

**Published:** 2019-05-28

**Authors:** Elias Torres Alonso, Dong‐Wook Shin, Gopika Rajan, Ana I. S. Neves, Saverio Russo, Monica F. Craciun

**Affiliations:** ^1^ Centre for Graphene Science, College of Engineering, Mathematics and Physical Sciences University of Exeter EX4 4QF Exeter UK

**Keywords:** complementary metal–oxide–semiconductors (CMOSs), graphene oxide, patterning, roll‐to‐roll, sensors, water‐exfoliated graphene

## Abstract

One of the main advantages of 2D materials for various applications is that they can be prepared in form of water‐based solutions. The high yield and cost‐effectiveness of this method make them of great interest for printed electronics, composites, and bio‐ and healthcare technologies. However, once deposited on a substrate, etching away these solution‐processed materials is a difficult task, yet crucial for pattern definition and thus device fabrication. In particular, the realization of micrometer‐sized patterns requires mesh and paste optimization when screen‐printed or solvent‐engineered and surface functionalization when inkjet‐printed, both usually involving additional postdeposition steps. These constraints are holding back the integration of these 2D materials in devices and applications. In this work, a method for the fabrication of micrometer‐sized well‐defined patterns in water‐based 2D materials is presented, with an extensive characterization of the films and patterns obtained. The method is ultimately used to create humidity sensors with performance comparable to that of commercial ones. These sensor devices are fabricated onto a 4′ silicon and polyethylene terephthalate (PET) wafers to create all‐graphene humidity sensors that are flexible, transparent, and compatible with current complementary metal–oxide–semiconductor (CMOS) and roll‐to‐roll workflows.

Monitoring humidity has become significant in many fields from electronic‐skin‐based diabetes therapy to industrial manufacturing. For example, sensing humidity is required for the proper functioning of diabetes patches,^1^ during the manufacturing processes of humidity‐sensitive products such as pulp, paper, and cardboard, and during packaging and transportation of vaccines, pharmaceuticals, food, and edibles in order to ensure the final quality of the products. For example, the World Health Organization requires that humidity monitoring systems should be used for time‐ and temperature‐sensitive pharmaceutical products (TTSPPs).^2^ Graphene as a representative element of the 2D materials family, with a remarkably high specific surface area‐to‐volume ratio, unique electrical,^3^ mechanical,^4^ and optical^5^ properties due to its soft carbon‐based nature, high carrier density,^6^ and ultrathin form factor, is one of the emerging materials for sensing applications, spanning from gas to light detection.^7^, ^8^, ^9^, ^10^, ^11^, ^12^, ^13^, ^14^, ^15^, ^16^, ^17^, ^18^, ^19^, ^20^, ^21^, ^22^, ^23^, ^24^, ^25^, ^26^, ^27^, ^28^, ^29^, ^30^, ^31^, ^32^, ^33^, ^34^ Particularly, graphene oxide (GO), a graphene derivative where oxygen‐containing functional groups, such as epoxy, hydroxyl, and carboxyl groups, cover the surface and edge,^14^ has been demonstrated as an excellent material for sensing humidity, benefiting from the hydrophilicity due to its dangling bonds.^15^, ^16^, ^17^, ^18^ Basically, highly defective GO with an insulator behavior has a high electrochemical activity, useful for various electrochemical sensors.^19^ Liquid exfoliation processes enable both graphene and GO as well as other 2D materials to be stably settled in various solvents,^20^, ^21^, ^22^, ^23^, ^24^ especially water exfoliations compatible to green and industrial technology. Their solutions can be exploited to fabricate humidity monitoring systems using many methods such as filtrating, spinning, spraying, and printing by screen and inkjet printing.

The scalability and cost‐effectiveness to produce solutions of 2D materials make them of big interest for the industry. Additionally, their production and deposition technologies, generally carried out at room or moderate temperatures, dramatically ease the integration of 2D materials with flexible polymeric substrates such as polyethylene terephthalate (PET) and polyethylene naphthalate (PEN) that are used massively as flexible electronic substrates. Indeed, recent developments of the deposition technologies of 2D solutions for flexible electronics have demonstrated vacuum filtration,^25^ roll‐to‐roll (R2R),^26^ inkjet,^25^, ^27^, ^28^, ^29^, ^30^ spray,^31^ and screen^32^ printing of graphene and inkjet printing of other 2D materials.^25^, ^28^, ^33^ Despite recent efforts, however, integrating these emerging 2D materials to omnipresent applications, especially humidity sensors, is still in infancy, and most of them do not fully exploit the 2D intrinsic features such as unprecedented mechanical flexibility, which will be of major importance when applications need to be embedded in consumer products. One of the main drawbacks to integrate these solutions into real‐life electronics is that once they are deposited as large area films, they are very difficult to pattern by means of physical and/or chemical etching, a key process for device fabrication. To overcome this issue, screen printing and R2R patterning processes without etching process have been provided, but it is really challenging to achieve patterns with linewidths of under 100 µm, and very extensive optimization of mesh (or screen masks) and paste formulation with additives such as polymeric resins are essentially required. Additionally, even though 2D materials' inkjet printing achieves micrometer‐sized resolution (10–100 µm) and functional devices, it is not suitable for large‐area printing with high throughput because of long process time; it requires relatively high‐temperature process as well as chemical and/or unsuitable thermal postdeposition treatments to achieve desired features.^25^, ^27^, ^28^, ^29^, ^30^, ^33^

Moreover, manufacturing devices integrated with liquid‐exfoliated 2D materials should be compatible with the existing approaches, especially with complementary metal–oxide–semiconductor (CMOS) technology, which is used ubiquitously for device processing, with no prospects to change in a short span of time. For a fast and successful integration of liquid‐exfoliated 2D materials into the semiconductor foundries, it is necessary to make use of existing CMOS techniques adapted to 2D materials, before specific processes may be gradually introduced into the production lines. Similarly, the emerging R2R processing is set to disrupt the way electronic devices are manufactured, especially fabrication of transparent and flexible electronics. This technique is already employed intensively to produce conductive patterns using Ag‐ and Cu‐based inks. The new properties achievable with the range of 2D inks available, suitable for cost‐effective production, will produce a much wider variety of devices fabricated by R2R, driving down material and manufacturing costs. Here, we demonstrate a workflow compatible with CMOS and R2R flexible technologies that allows 4′ wafer‐scale integration of both graphene and graphene oxide inks, specifically all‐graphene humidity sensor array. We employed water‐exfoliated graphene and GO solutions to create all‐graphene humidity sensors integrated with interdigitated graphene electrodes and thin GO films as sensing layer over wafer scale, with CMOS and R2R compatibility and room‐temperature processing techniques. This new technique allows the accurate placing and deposition of solution‐processed graphene and graphene oxide in a large scale without the etching process, overcoming one of the main issues of these solutions. These sensors have uniform response, good lifetime, transparency, and flexibility. In principle, this method can be conveniently adapted to any kind of 2D material dispersion to create patterns and devices with different functionalities with high yield over large areas.

To illustrate the technique for the fabrication of all‐graphene humidity sensors, we have schematized the process in **Figure** **1**a. First, layers of polymethylglutaramide (PMGI) with ≈250 nm and photoresist (PR) with ≈1.4 µm are spun and baked over a Si/SiO_2_ substrate (see the details in the “Experimental Section”). Then, PR is exposed with an UV source along a digital input pattern mask to create a pattern, and is then developed. As a pattern, we demonstrate here an interdigitated electrode geometry, which is the most widely accepted geometry for the electrodes of a sensor as it enables a wide contact area between the electrodes within a limited sensor area. The interdigitated PR patterns were obtained for the electrodes of the humidity sensor and identification of conductivity and fine deposition of graphene, respectively, as shown in Figure 1b. The developer dissolves not only the areas where the PR has been exposed (acting as an image resist), but also to the PMGI layer which is underneath. After the development step, the PR is stripped off with acetone and isopropanol (IPA), whereas the patterned PMGI remains. Subsequently, the deposition of water‐exfoliated graphene (prepared by shear exfoliation technique^20^ using sodium cholate as surfactant) takes place using an IPA‐assisted direct transfer (IDT) method developed previously in our group.^35^ Water‐exfoliated graphene solution was filtrated onto hydrophilic polytetrafluoroethylene (PTFE) filters using a vacuum filtration system, and the graphene film formed on the membrane filter was readily transferred by IDT^35^ onto the substrate with the patterned PMGI. Several sequential transfers can be performed to increase conductivity and film uniformity. The PMGI is dissolved in *N*‐methyl‐2‐pyrrolidone (NMP) under mild ultrasonication (lift‐off process), resulting in the fine patterned graphene film (optical microscope image of Figure 1c). This selective deposition process without physical and chemical etching processes is then repeated for the GO, which is used as the layer‐sensing humidity deposited on top of the interdigitated graphene electrodes (Figure 1d). The vacuum‐filtrated GO on a cellulose filter membrane is transferred on the graphene electrodes under N_2_ dry blowing. Finally, a metallization step, consisting of the deposition by thermal evaporation of a 5 nm of Cr as a wetting layer and 90 nm of Au on both ends of the interdigitated graphene electrode not covered by GO film, is added to reduce a contact resistance between probes and graphene electrodes on humidity devices (1 cm^2^ Si/SiO_2_ chip; Figure 1e).

**Figure 1:**
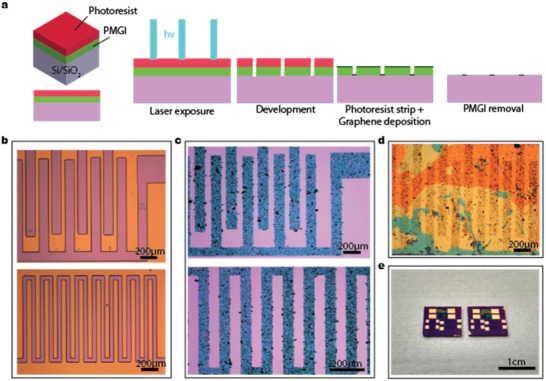
a) Schematic of the process. b) Optical microscope image of the interdigitated electrodes (top) and snake‐like structures (bottom). c) Optical microscope image of the resulting graphene pattern. d) Optical microscope image after graphene oxide deposition. e) Photograph of finished devices after metallization.

In order to determine the resolution of our patterning and deposition technique, lines ranging from 20 µm up to 200 µm (gap of lines from 5 to 100 µm) were patterned, as shown in **Figure** **2**a (see Figure S1 in the Supporting Information). It was found that at least 50 µm wide was the minimal width where graphene could be deposited with an acceptable coverage, comparable to those features achieved by inkjet printing.^25^, ^27^, ^28^, ^29^, ^30^ This coverage can be improved realizing successive depositions by the IDT method. To assess this step, we patterned 100 µm lines and performed a series of 2, 4, and 6 subsequent depositions (2Ds, 4Ds, and 6Ds; Figure S2, Supporting Information). Using 2Ds rendered poor graphene coverage and no conductive patterns. Over 4Ds, we obtained conductive patterns, and their conductivity changed very little with increasing the number of subsequent depositions. We performed profile scans with an atomic force microscope (AFM). In Figure 2b,c, the AFM images are shown for graphene using 4Ds and for GO, respectively. Multiple scans were performed to average a value of thickness of such films, resulting in an averaged thickness of 215 nm for 4Ds and 316 nm for 6Ds (Figure S3, Supporting Information), with a mean square roughness (RMS) of 78.72 and 108 nm respectively. In the AFM images, one can see the well‐defined edges of the graphene tracks, and also appreciate their rough surface (inset of Figure 2b). In the case of GO, as shown in Figure 2c, the observed thickness was 31 nm with a much smoother surface of only 9 nm of RMS. A Raman map was taken, with a 2 µm pitch in between spots. The number of layers was established using a previously published method by Paton et al.^20^ The Raman map in Figure 2d shows an average of six layers; comparing it with the thickness of the films derived from AFM measurements and assuming 2 nm thickness of a single layer encapsulated by sodium cholate and 1 nm of interlayer spacing,^20^ we estimated that the films are composed of an average number of 30 and 45 graphene flakes of six layers for the 4D and 6D samples, respectively. Additionally, we characterized the identification and crystalline quality of liquid‐exfoliated graphene and GO by X‐ray diffraction (XRD) and Raman, as shown in Figure 2e,f. In the graphene, we observed a high crystalline quality of its film by XRD, with the 26.6° peak present, which has the same position in graphite. In addition, a high crystalline quality of liquid‐exfoliated graphene (shear exfoliated) was also demonstrated by our recent work using X‐ray photoelectron spectroscopy (XPS) and Raman.^35^ In GO, we observed a 10.9° peak and a large D‐band and broaden G‐band in the XRD and Raman spectra, respectively. This is typically observed in GO film due to a high density of oxygen‐containing functional groups on the surface and edge of graphene.^14^, ^19^

**Figure 2:**
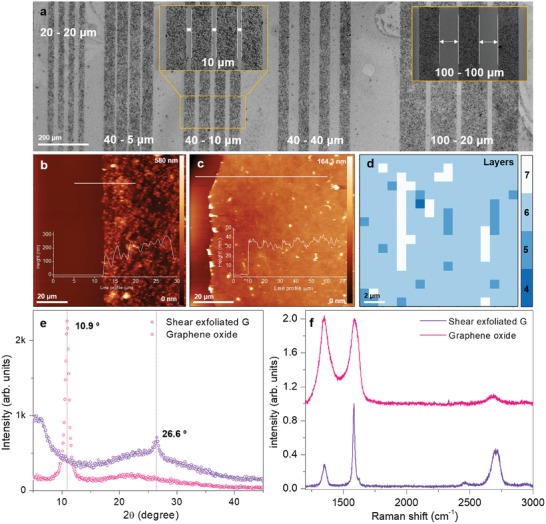
a) Resolution of line patterns of liquid‐exfoliated graphene formed by our deposition technique. The size of line patterns, observed from an SEM image, is (20 (line width) –20 (gap between lines), 40–5, 40–10, 40–40, 100–20, and 100–100 µm. AFM image of b) liquid‐exfoliated graphene and c) graphene oxide used in the humidity sensor and their thickness (inset). d) Raman map of the liquid‐exfoliated graphene film. e) XRD and f) Raman spectra of liquid‐exfoliated graphene and graphene oxide.

Benefiting from the deposition and pattering methods demonstrated above, graphene humidity sensors were fabricated, and their performance was analyzed. The physical process giving rise to this response is summarized as follows: in GO, planes and edges contain oxygen functional groups, which gives GO its hydrophilicity and also their naturally insulating properties.^14^ As relative humidity (RH) increases, the water molecules will undergo physisorption by GO. This physisorbed water molecules can be ionized producing hydronium ions (H_3_O^+^) that behave as charge carriers.^18^, ^36^ As more water is physisorbed, proton‐transfer reactions take place, hopping in between water molecules and oxygen‐rich groups within the GO via Grotthuss chain reaction.^18^, ^36^ This, in turn, increases the conductance which is used as sensing parameter. **Figure** **3**a shows the change in conductance produced when the samples are exposed to humidity resulting from human breath, and it is compared to that of a commercially available CMOS capacitive sensor. The performance is very similar, while the minute changes in response may be attributed to a slightly different positions of the samples, being affected differently by different air flows; nevertheless, the correlation between our all‐graphene device and the CMOS sensor is clear. Moreover, this change in conductance is mainly due to a change in electrical resistance, with a negligible capacitive change, which simplifies the electronics for device readout. The excitation voltage was only 1 V, suitable for low‐power electronics. We tested our device in very humid atmospheres, up to 97% RH; the performance is similar to that of the commercial sensor, as seen in Figure 3b. We also observe no correlation in the temperature and humidity response for our device, which should ease integration into devices for applications. In Figure 3c, we show the temperature dependence of the conductance from room temperature up to 55 °C, with no appreciable change in conductance observed. Graphene oxide undergoes a thermally driven phase transformation upon heating at mild temperatures (i.e., 80 °C), which would, in fact, reduce its sensing properties.^37^ To assess this, we evaluated the response of the device after heating it up to 80 °C. The sensor response after heating, shown in Figure 3d, remains unaltered. Moreover, the reproducibility of the process is also assessed comparing four different devices, two processed on the same chip and two processed in a different chip. Their response upon exposure to human breath and to high humidity is found to be very similar, confirming the reliability of the technique for batch processing (see Figures S4 and S5 in the Supporting Information). Remarkably, the performance of these sensors remains reliable after 90 days of storage in ambient conditions (see Figure S6 in the Supporting Information), which is at least three times longer than previously reported GO‐based devices.^17^, ^18^

**Figure 3:**
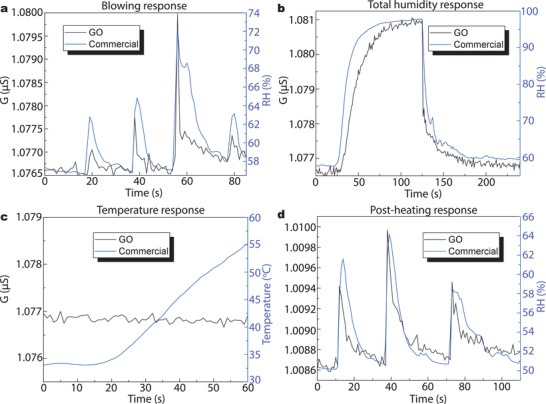
a) Device response to human blowing. b) Device response to a large humidity change. c) Device response to a temperature change. d) Device response to human blowing after an annealing step of 60 °C for 5 min.

We mentioned before that for the successful incorporation of the 2D materials into the industries, the workflow needs to be adapted to their requirements such as the implementation of mass production and reproducibility (high yield). We note that all the processes carried out so far are compatible or have potential to be introduced into CMOS back‐end production line (BEPL). To assess this, we processed a 4′ Si/SiO_2_ wafer where we created devices of 1 cm^2^ as shown in **Figure** **4**a. A typical device response among all the devices is shown in Figure 4b; the difference in time response can be again attributed to different position of the two different sensors. Contacts were made with 100 nm thick Al layer, since Au is not suitable for CMOS due to introduction of midgap states in Si. Furthermore, due to the low‐temperature processing, we were able to build sensors on PET substrates, which have great transparency and flexibility as shown in Figure 4c,d. In Figure 4c, we created fully functional devices with a footprint of just 25 mm^2^, contacted with a thin wetting layer of Cr (5 nm) and thick layer (100 nm) of Au; in Figure 4d, 1 cm^2^ devices were created and contacted with commercial carbon paste, creating all‐carbon‐based humidity sensors. The performance of these devices before and after 2000 bending cycles (5 mm radius) is shown in Figure 4e; it remains identical, demonstrating the good mechanical properties of our devices.

**Figure 4:**
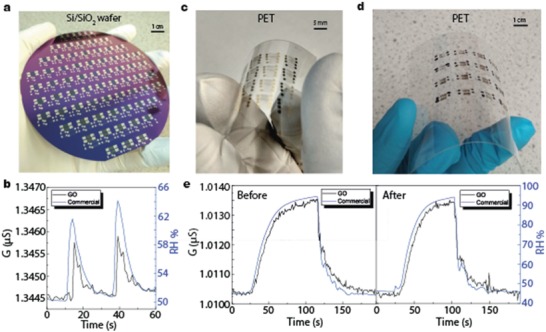
a) Photograph of a 4′ wafer of Si/SiO_2_ with several devices. b) Response to human blowing from a representative device of the 4′ wafer of Si/SiO_2_. All‐graphene devices built on PET substrate with c) Cr/Au contacts and d) with carbon paste contacts (all‐carbon devices). e) Device response before (left) and after (right) 2000 bends. This performance corresponds to the device seen in panel (b).

Finally, we assessed the sensitivity of our devices as 0.2%, calculated as *S* = (*G*_90_ − *G*_40_)/*G*_40_ = 0.2%,^18^ where *G_x_* is the conductance at an *x* value of %RH, in our case 90% (top) and 40% (bottom) humidity. As opposed to other sensors, the range of sensitivity of our sensors is of interest for low power, Internet of Things (IoT) applications, where simple electronics are needed, which may not cover a range of signals in the range of thousands of microvolts. Moreover, the voltage supply employed in our device was 1 V, with an estimated current of 1 µA, which gives a power of 1 µW for normal performance. For comparison, the CMOS sensor needs 200 µA at 5 V supply, which translates into a powering need of 1 mW, i.e., three orders of magnitude more power required than in the case of our sensors.

In conclusion, this work reports a combination of techniques that allowed to accurately create patterns and deposit solution‐processed 2D materials such as graphene and GO. We would like to emphasize that, after parameter optimization, this process should be compatible with any type of 2D materials, opening up ways to construct functional yet cost‐effective, flexible, fully functional electronics with these materials. Several transfers can be performed to increase conductivity and film uniformity. Stacks of different materials and films could potentially be made using this technique, and at least four depositions are necessary to create graphene patterns that are conductive in our work. In the polymer‐free process, the final 2D materials are never in touch with a polymer, reducing the well‐known polymeric contamination. The potential of the process is further demonstrated by comparing the performance of four different devices and showing that their performances are very similar and correlate very well with that of a commercially available sensor. Finally, the scalability of this process was shown creating a whole 10′ Si/SiO_2_ wafer with exceptional CMOS compatibility; we also employed this process to create all‐graphene flexible, transparent devices on PET substrate for the first time, which are also resilient to intense mechanical stresses. We strongly believe that this technique may become a cornerstone when integrating solution‐processed 2D materials into real‐world, CMOS‐based applications in the future, and we forecast massive potential for applications where cost‐effective and disposable electronics are needed.

## Experimental Section

*Lithography*: PMGI SF6 (from MicroChem Corp.) was spun at 4000 rpm, baked at 180 °C for 360 s. After then, photoresist S1813 (MicroChem Corp.) was spun at 4000 rpm and baked at 120 °C. Resists (S1813 (top)/PMGI (bottom)) deposited on desired substrates were exposed in a laser‐writer (Durham Magnetoptics Ltd) by UV light along the digital input pattern mask to create a pattern, and exposed S1813 resist was developed in an alkaline‐based solvent (MF‐319 from MicroChem Corp) as well as at the same time, the PMGI layer underneath was etched by the same solvent along the S1813 resist pattern. Then, the S1813 resist was stripped off with acetone and IPA.

*Graphene Solutions and Films*: The solution was created with a concentration of 15 mg mL^−1^ for graphite powder from Sigma‐Aldrich and 5 mg mL^−1^ of sodium cholate from Sigma‐Aldrich mixed in 800 mL of de‐ionized water (DIW). This solution was mixed with a high shear mixer from Silverson for 60 min at 4500 rpm. They were then centrifuged for 60 min at 1500 rpm and decanted. This solution was additionally dipped in an ultrasonic bath for 90 min, and further centrifuged for 90 min and then decanted to remove unwanted thick graphene flakes. About 10 mL of this solution was dispersed in 60 mL of DIW and vacuum‐filtered on a PTFE membrane from Millipore, which was finally used for the IDT.

*IPA‐Assisted Direct Transfer*: The sample with the PMGI pattern was placed on a hotplate at 90 °C, covered with IPA, and immediately after the membrane was placed on top of the sample, with the graphene facing down. After ≈300 s, all the IPA was evaporated and the membrane was removed. This step was repeated after desired films were deposited.

*Characterization of Patterned Graphene and GO*: Raman mapping was performed using a RM1000 from Renishaw with a 532 nm laser source and air‐cooled charge‐coupled detectors. The spot size was ≈1 µm and the pitch was 2 µm to ensure no overlap between scans. The number of graphene layers was extracted by the method that was developed by Paton et al.^20^ Contact‐height mode scans were performed with an AFM Innova from Bruker Ltd. to measure the thickness of patterned graphene and GO in the humidity sensor. Scanning electron microscopy (SEM) images of line patterns of graphene were taken with a Hitachi S3200N SEM with an acceleration voltage of 20 keV.

*Humidity, Temperature, and Conductance Measurements*: RH was measured using a HIH‐4000‐003 humidity sensor from Honeywell; temperature was measured using a TMP36GT9Z‐ND temperature sensor from Digi‐Key Electronics. They were both powered with 5 V using a 2400 power source from Keithley, and voltage drops were recorded with a 34 401 multimeter from Agilent. Graphene/GO sensors conductance were recorded with an HM8118 LCR bridge from Rohde and Schwarz at 1 kHz and 0.5 V. The data were collected using LabView software from National Instruments.

## Conflict of Interest

The authors declare no conflict of interest.

## Supporting information

Supplementary

## References

[1] H. Lee , T. K. Choi , Y. B. Lee , H. R. Cho , R. Ghaffari , L. Wang , H. J. Choi , T. D. Chung , N. Lu , T. Hyeon , S. H. Choi , D.‐H. Kim , Nat. Nanotechnol. 2016, 11, 566. 26999482 10.1038/nnano.2016.38

[2] World Health Organization , W. H. O. Tech. Rep. Ser. 2011, 961, 342. 22397172

[3] K. I. Bolotin , K. J. Sikes , Z. Jiang , M. Klima , G. Fudenberg , J. Hone , P. Kim , H. L. Stormer , Solid State Commun. 2008, 146, 351.

[4] C. Lee , X. Wei , J. W. Kysar , J. Hone , Science 2008, 321, 385. 18635798 10.1126/science.1157996

[5] R. R. Nair , P. Blake , A. N. Grigorenko , K. S. Novoselov , T. J. Booth , T. Stauber , N. M. R. Peres , A. K. Geim , Science 2008, 320, 1308. 18388259 10.1126/science.1156965

[6] R. Murali , Y. Yang , K. Brenner , T. Beck , J. D. Meindl , Appl. Phys. Lett. 2009, 94, 243114.

[7] F. Schedin , A. K. Geim , S. V. Morozov , E. W. Hill , P. Blake , M. I. Katsnelson , K. S. Novoselov , Nat. Mater. 2007, 6, 652. 17660825 10.1038/nmat1967

[8] H. J. Yoon , D. H. Jun , J. H. Yang , Z. Zhou , S. S. Yang , M. M.‐C. Cheng , Sens. Actuators, B 2011, 157, 310.

[9] Y. Dan , Y. Lu , N. J. Kybert , Z. Luo , A. T. Charlie Johnson , Nano Lett. 2009, 9, 1472. 19267449 10.1021/nl8033637

[10] T. Mueller , F. Xia , P. Avouris , Nat. Nanotechnol. 2010, 4, 297. 10.1038/nnano.2009.292 19893532

[11] F. Xia , T. Mueller , Y. Lin , A. Valdes‐Garcia , P. Avouris , Nat. Photonics 2009, 4, 839. 10.1038/nnano.2009.292 19893532

[12] X. Gan , R.‐J. Shiue , Y. Gao , I. Meric , T. F. Heinz , K. Shepard , J. Hone , S. Assefa , D. Englund , Nat. Photonics 2013, 7, 883.

[13] E. W. Hill , A. Vijayaragahvan , K. Novoselov , K. Graphene , Sensors 2011, 11, 3161.

[14] D. R. Dreyer , S. Park , C. W. Bielawski , R. S. Ruoff , Chem. Soc. Rev. 2010, 39, 228. 20023850 10.1039/b917103g

[15] J. T. Robinson , F. K. Perkins , E. S. Snow , Z. Wei , P. E. Sheehan , Nano Lett. 2008, 8, 3137. 18763832 10.1021/nl8013007

[16] G. Lu , L. E. Ocola , J. Chen , Nanotechnology 2009, 20, 455502. 19809107 10.1088/0957-4484/20/44/445502

[17] S. Borini , R. White , D. Wei , M. Astley , S. Haque , E. Spigone , N. Harris , J. Kivioja , T. Ryhänen , ACS Nano 2013, 7, 11166. 24206232 10.1021/nn404889b

[18] H. Bi , K. Yin , X. Xie , J. Ji , S. Wan , L. Sun , M. Terrones , M. S. Dresselhaus , Sci. Rep. 2013, 3, 2714. 24048093 10.1038/srep02714 PMC3776968

[19] C. Chung , Y.‐K. Kim , D. Shin , S.‐R. Ryoo , B. H. Hong , D.‐H. Min , Acc. Chem. Res. 2013, 46, 2211. 23480658 10.1021/ar300159f

[20] K. R. Paton , E. Varrla , C. Backes , R. J. Smith , U. Khan , A. O'Neill , C. Boland , M. Lotya , O. M. Istrate , P. King , T. Higgins , S. Barwich , P. May , P. Puczkarski , I. Ahmed , M. Moebius , H. Pettersson , E. Long , J. Coelho , S. E. O'Brien , E. K. McGuire , B. M. Sanchez , G. S. Duesberg , N. McEvoy , T. J. Pennycook , C. Downing , A. Crossley , V. Nicolosi , J. N. Coleman , Nat. Mater. 2014, 13, 624. 24747780 10.1038/nmat3944

[21] F. Bonaccorso , A. Lombardo , T. Hasan , Z. Sun , L. Colombo , A. C. Ferrari , Mater. Today 2012, 15, 564.

[22] J. N. Coleman , M. Lotya , A. O'Neill , S. D. Bergin , P. J. King , U. Khan , K. Young , A. Gaucher , S. De , R. J. Smith , I. V. Shvets , S. K. Arora , G. Stanton , H.‐Y. Kim , K. Lee , G. T. Kim , G. S. Duesberg , T. Hallam , J. J. Boland , J. J. Wang , J. F. Donegan , J. C. Grunlan , G. Moriarty , A. Shmeliov , R. J. Nicholls , J. M. Perkins , E. M. Grieveson , K. Theuwissen , D. W. McComb , P. D. Nellist , V. Nicolosi , Science 2011, 331, 568. 21292974 10.1126/science.1194975

[23] Y. Wei , Z. Sun , Curr. Opin. Colloid Interface Sci. 2015, 20, 311.

[24] V. Nicolosi , M. Chhowalla , M. G. Kanatzidis , M. S. Strano , J. N. Coleman , Science 2013, 340, 1226419.

[25] F. Withers , H. Yang , L. Britnell , A. P. Rooney , E. Lewis , A. Felten , C. R. Woods , V. Sanchez Romaguera , T. Georgiou , A. Eckmann , Y. J. Kim , S. G. Yeates , S. J. Haigh , A. K. Geim , K. S. Novoselov , C. Casiraghi , ACS Nano 2014, 14, 3987. 10.1021/nl501355j 24871927

[26] http://www.cam.ac.uk/research/news/new‐graphene‐based‐inks‐for‐high‐speed‐manufacturing‐of‐printed‐electronics (accessed: December 2018).

[27] S. Santra , G. Hu , R. C. T. Howe , A. De Luca , S. Z. Ali , F. Udrea , J. W. Gardner , S. K. Ray , P. K. Guha , T. Hasan , Sci. Rep. 2015, 5, 17374. 26616216 10.1038/srep17374 PMC4663628

[28] a) D. McManus , S. Vranic , F. Withers , V. Sanchez‐Romaguera , M. Macucci , H. Yang , R. Sorrentino , K. Parvez , S. K. Son , G. Iannaccone , K. Kostarelos , G. Fiori , C. Casiraghi , Nat. Nanotechnol. 2017, 12, 343; 28135260 10.1038/nnano.2016.281

[29] a) K.‐Y. Shin , J.‐Y. Hong , J. Jang , Adv. Mater. 2011, 23, 2113; 21484894 10.1002/adma.201100345

[30] a) F. Torrisi , T. Hasan , W. Wu , Z. Sun , A. Lombardo , T. S. Kulmala , G.‐W. Hsieh , S. Jung , F. Bonaccorso , P. J. Paul , D. Chu , A. C. Ferrari , ACS Nano 2012, 6, 2992; 22449258 10.1021/nn2044609

[31] S. C. Patel , O. Alam , D. Zhang , K. Grover , Y.‐X. Qin , B. Sitharaman J. Mater. Res. 2017, 32, 370.

[32] K. Arapov , E. Rubingh , R. Abbel , J. Laven , G. de With , H. Friedrich , Adv. Funct. Mater. 2016, 26, 586.

[33] a) D. J. Finn , M. Lotya , G. Cunningham , R. J. Smith , D. McCloskey , J. F. Donegana , J. N. Coleman , J. Mater. Chem. C 2014, 2, 925;

[34] J. D. Mehew , S. Unal , E. Torres Alonso , G. F. Jones , S. F. Ramadhan , M. F. Craciun , S. Russo , Adv. Mater. 2017, 29, 1700222. 10.1002/adma.201700222 28418620

[35] D.‐W. Shin , M. D. Barnes , K. Walsh , D. Dimov , P. Tian , A. I. S. Neves , C. D. Wright , S. M. Yu , J.‐B. Yoo , S. Russo , M. F. Craciun , Adv. Mater. 2018, 30, 1802953. 10.1002/adma.201802953 30141202

[36] N. Agmon , Chem. Phys. Lett. 1995, 244, 456.

[37] P. V. Kumar , N. M. Bardhan , S. Tongay , J. Wu , A. M. Belcher , J. C. Grossman , Nat. Chem. 2014, 6, 151. 24451592 10.1038/nchem.1820

