# Supplementary material for: Water‐Based Solution Processing and Wafer‐Scale Integration of All‐Graphene Humidity Sensors
Source: Adv Sci (Weinh). 2019 May 28;6(15):1802318. doi: 10.1002/advs.201802318 (PMC6685499; doi:10.1002/advs.201802318)
Supplement: Supplementary file 1 — Supplementary [file ADVS-6-1802318-s001.pdf]

Copyright WILEY-VCH Verlag GmbH & Co. KGaA, 69469 Weinheim, Germany, 2019.

ADVANCED SCIENCE
Open Access

Supporting Information

for *Adv. Sci.,* DOI: 10.1002/advs.201802318

Water-Based Solution Processing and Wafer-Scale Integration of All-Graphene Humidity Sensors

*Elias Torres Alonso, Dong-Wook Shin, Gopika Rajan, Ana I. S. Neves, Saverio Russo, and Monica F. Craciun**

Copyright WILEY-VCH Verlag GmbH & Co. KGaA, 69469 Weinheim, Germany, 2018.

Supporting Information

Supporting Information for "Wafer-scale integration of water-based 2D inks for all-carbon humidity sensors"

*Elias Torres Alonso, Dong-Wook Shin, Gopika Rajan, Ana I. S. Neves, Saverio Russo and Monica Craciun**

School of Physics, Stocker Road, EX4 4QL, Exeter, UK.
E-mail: M.F.Craciun@exeter.ac.uk

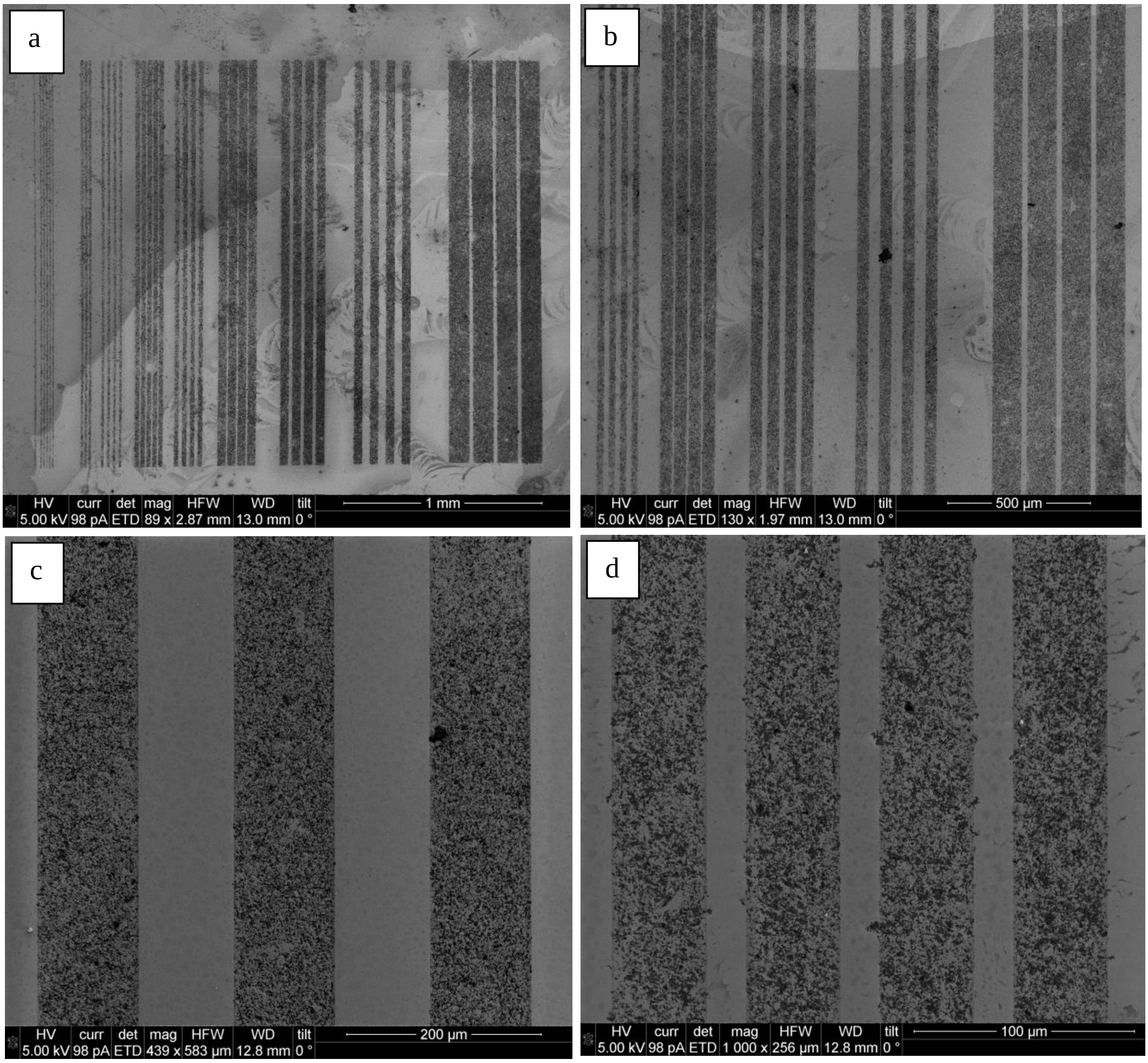

Figure S1: a) Deposition test with lines ranging from 10 µm to 100 µm wide and pitch from 20 µm to 50 µm. b) Magnified image showing lines of (from left to right) 20 µm wide with 20 µm pitch, 40 µm wide with 5 µm pitch, 40 µm wide with 10 µm pitch, 40 µm wide with 40 µm pitch and 100 µm wide with 20 µm pitch. c) Magnified image of 100 µm wide with 100 µm pitch. d) Magnified image of 50 µm lines with 20 µm pitch in between lines. All the images are after 6 graphene depositions.

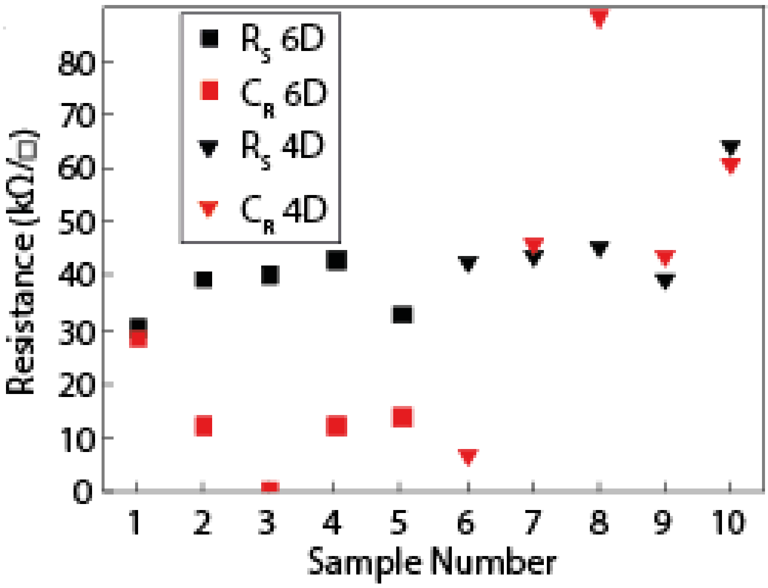

Figure S2. Mutiple transfers of liquied exfoliated graphene (2Ds, 4Ds and 6Ds). Only 2 deposition rendered poor graphene coverage and no conductive patterns. At 4Ds we achieved an electrical conductance of graphene electrode as a threshold of percolation path. At 6Ds, we obtained electrically enhnaced graphene electrode due to the increase of graphene density in the film. Even though the values of sheet resistance are similar for both 4D (~ 46kΩ/□) and 6D (~ 37kΩ/□), we observed at 6Ds there was less contact resistance (14 kΩ/□). This difference in contact resistance is probably related with the larger graphene coverage achieved with 6D than with 4D.

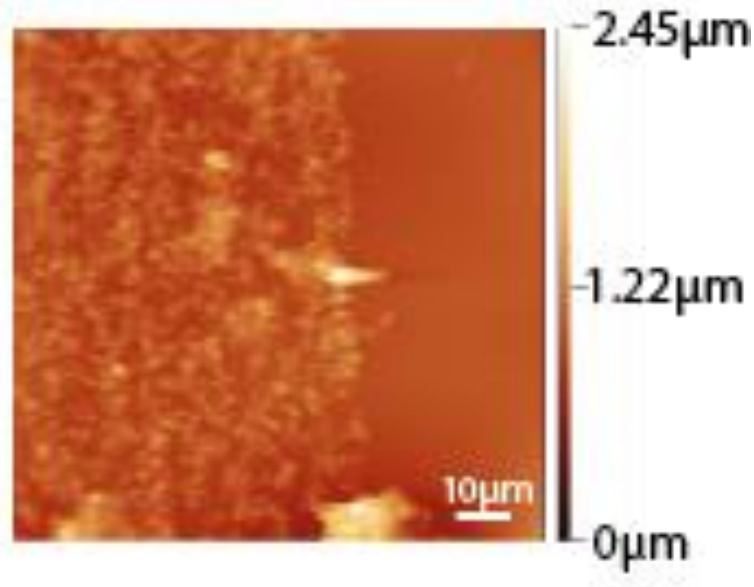

Figure S3. AFM image of graphene by 6Ds

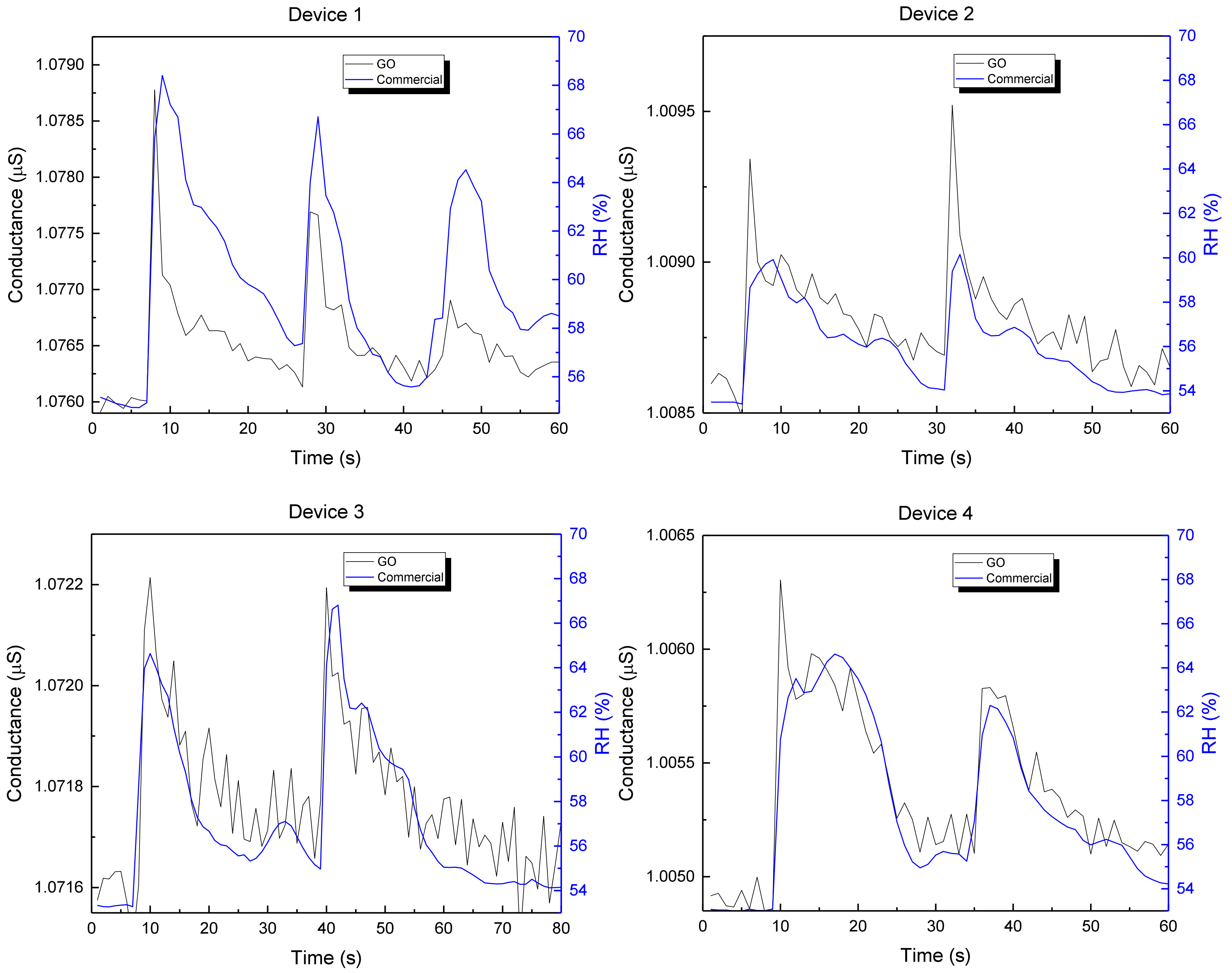

Figure S4. Response of 4 different devices under human blowing. Devices 1 and 2 were built in the same chip, and devices 3 and 4 were built in the same chip.

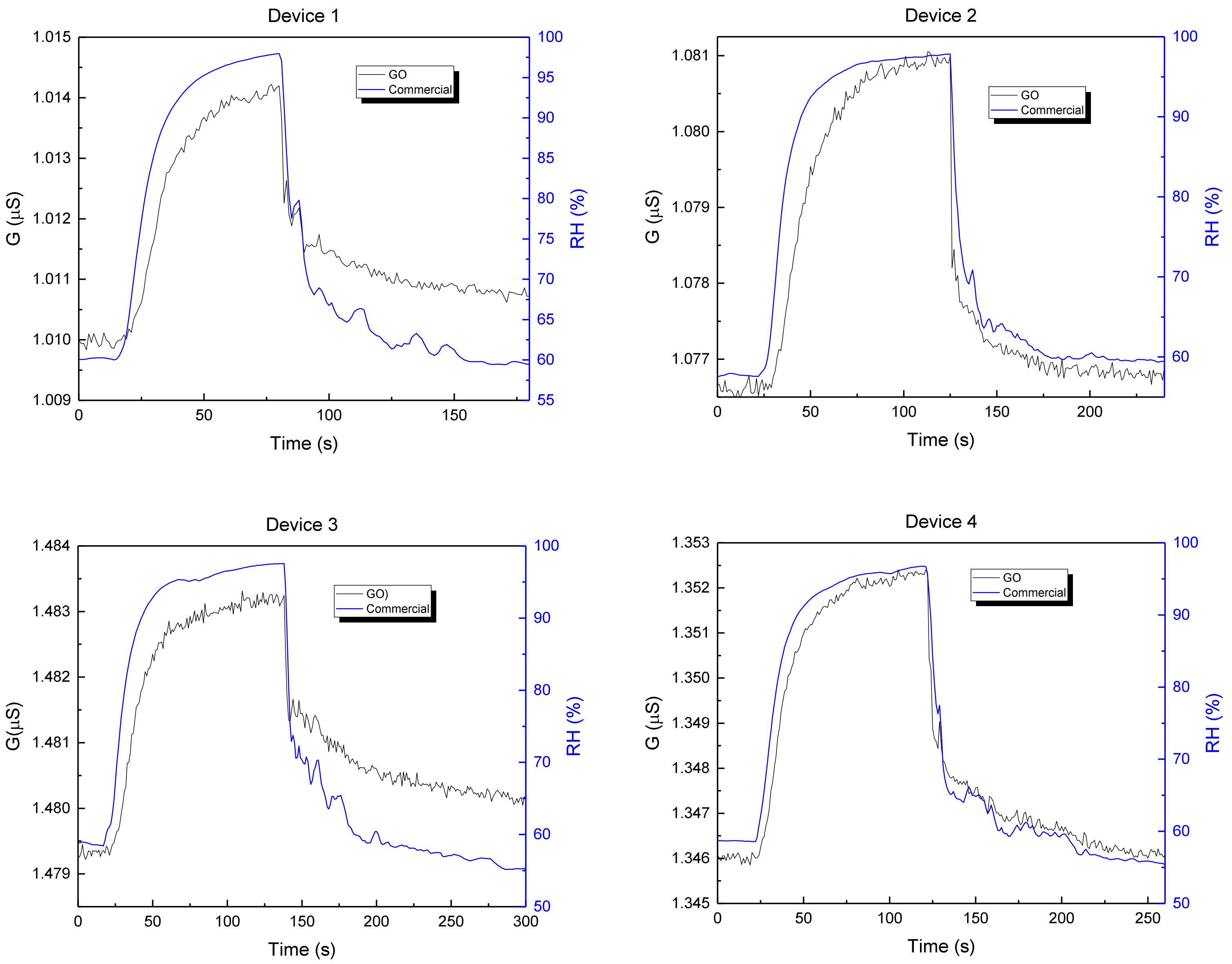

Figure S5. Response of 4 different devices under high humidity environment. Devices 1 and 2 were built in the same chip, and devices 3 and 4 were built in the same chip.

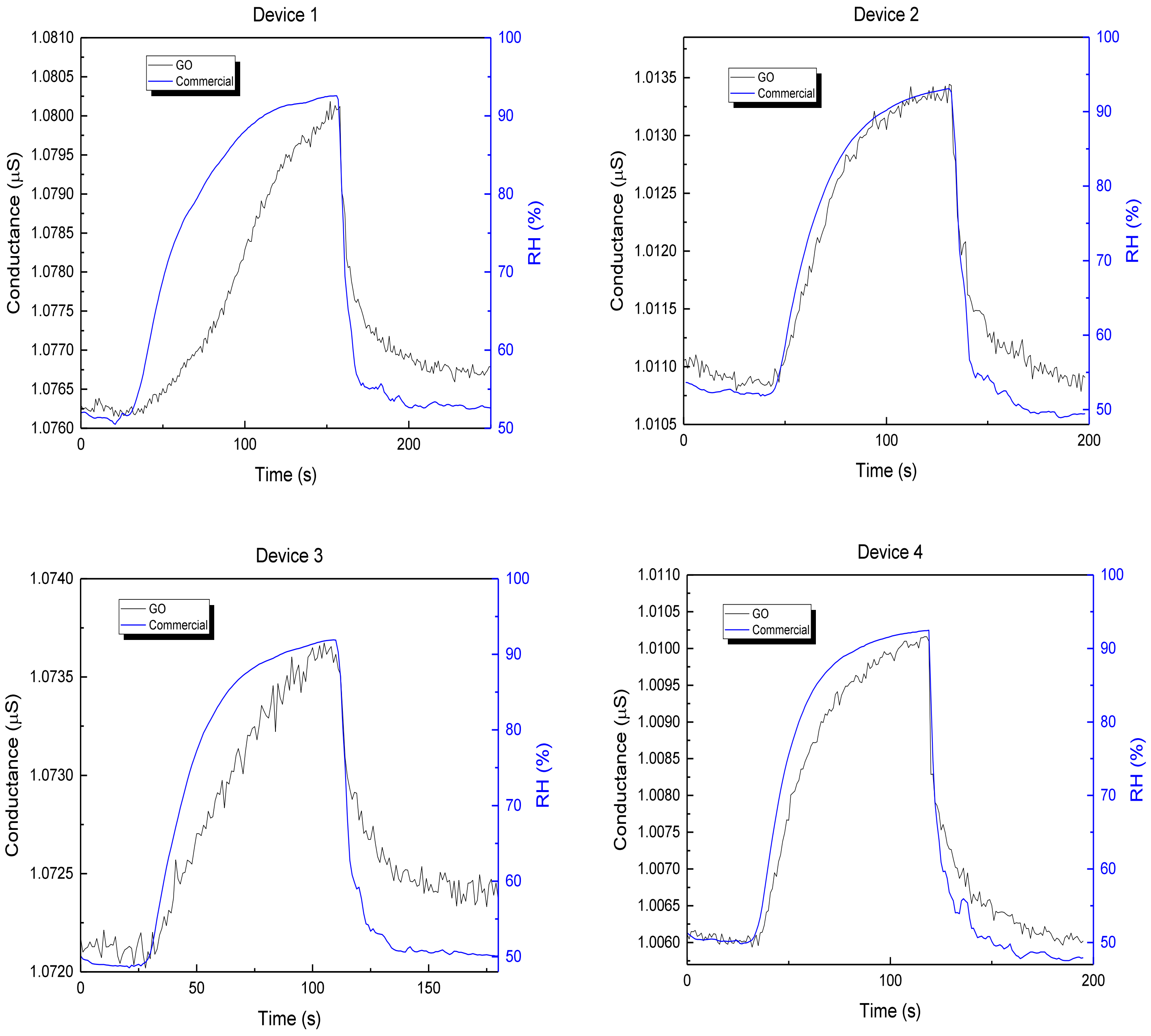

Figure S6. Response of 4 different devices under high humidity environment after 90 days of storage in ambient conditions. Devices 1 and 2 were built in the same chip, and devices 3 and 4 were built in the same chip.

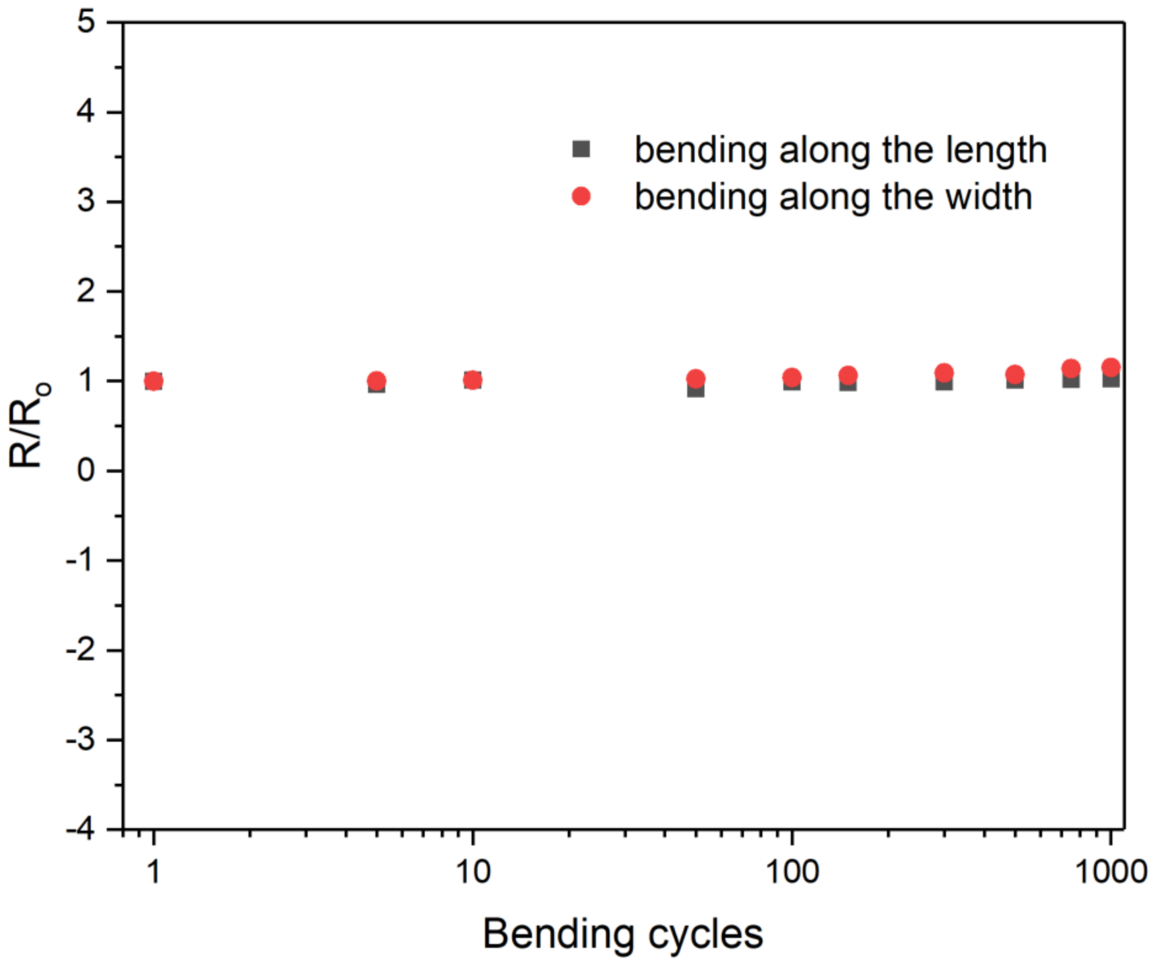

Figure S7. Flexibility studies under different deformation tests that show the relative changes in the resistance of the liquied exfoliated graphene used for the contacts of the sensor as a function of bending cycles when the device is exposed to different deformation states.
